# Primary prophylaxis of hepatic encephalopathy in decompensated cirrhosis: Low dose vs. full dose rifaximin

**DOI:** 10.12669/pjms.35.5.549

**Published:** 2019

**Authors:** Shahid Sarwar, Bushra Muhyuddin, Abdul Aleem, Muhammad Arif Nadeem

**Affiliations:** 1 Shahid Sarwar, MBBS, FCPS (Medicine) FCPS (Gastroenterology), MCPS-HPE, FRCP (Edin). Associate Professor of Medicine, Services Institute of Medical Sciences (SIMS), Lahore, Pakistan; 2 Bushra Muhyuddin, MBBS, FCPS (Medicine). Senior Registrar, Services Hospital, Lahore, Pakistan. Services Institute of Medical Sciences (SIMS), Lahore, Pakistan; 3 Abdul Aleem, MBBS. Resident Gastroenterology, Services Hospital, Lahore, Pakistan. Services Institute of Medical Sciences (SIMS), Lahore, Pakistan; 4 Muhammad Arif Nadeem, MBBS, FCPS (Medicine). Professor of Medicine, Services Institute of Medical Sciences (SIMS), Lahore, Pakistan

**Keywords:** Decompensated cirrhosis, Portosystemic encephalopathy, Primary prophylaxis, Rifaximin

## Abstract

**Objective:**

To compare efficacy of high vs low dose rifaximin for primary prophylaxis of portosystemic encephalopathy (PSE) in decompensated liver cirrhosis.

**Methods:**

In a quasi-experimental double blind randomized study at Services Institute of Medical Sciences (SIMS), Lahore from August 2017 to August 2018, patients of decompensated cirrhosis with no previous PSE were randomized to receive twice daily rifaximin 200mg in Group-A and 550mg in Group-B. Patients were followed for 6 months for development of PSE.

**Results:**

In 75 included patients, mean age was 53.8(±10.7) years and male/female ratio was 0.97/1(37/38). After randomization, 34 (45.3%) patients were included in Group-A and 41 (54.7%) patients in Group-B. During 6 month follow up 24 (32%) patients developed PSE, 12 (35.2%) in Group-A and 12 (29.2%) in Group-B, difference was not significant (p value 0.57). In 6 months, 13 (17.3%) patient died, 6 (17.6%) in Group-A and 7 (17.07%) patients in Group-B, difference not significant (p value 0.94). Patients who died had higher bilirubin (p < 0.00), higher serum creatinine (p 0.05), high CTP score (p 0.04) and worse MELD score (p 0.004).

**Conclusion:**

Rifaximin is not effective for primary prophylaxis of overt hepatic encephalopathy in decompensated cirrhosis patients.

## INTRODUCTION

Hepatic encephalopathy is a neuropsychiatric disorder seen in patients with liver cirrhosis.^1^ Development of encephalopathy in a cirrhosis patient is associated with high mortality and bad prognosis.^2^

Hepatic encephalopathy is the consequence of failure of liver to detoxify various toxins circulating in body due to impaired liver functions.^3^ Accumulation of these toxins mainly ammonia results in passage of these harmful substances across blood brain barrier. Ammonia is converted in to glutamate in astrocytes resulting in cerebral edema and altered sensorium.^4^

Majority of these toxins including ammonia are produced in intestine by bacterial fermentation of dietary protein. Once absorbed in circulation, these toxins are free to produce complications like hepatic encephalopathy due to insufficiency of hepatic metabolism.^5^

Due to this pathophysiology, treatment of hepatic encephalopathy is focused on reducing ammonia production in gut by controlling amount of dietary protein intake, laxative use to reduce stay time of undigested dietary protein and suppressing ammonia producing bacteria in gut by using disaccharides and antibiotics.^3^

Rifaximin is a poorly absorbed (0.4%) antibiotic which is effective against broad range of gram positive, gram negative aerobes and anaerobes.^6^ It has shown to be effective in treating as well as reducing recurrence of hepatic encephalopathy in liver cirrhosis and is presently recommended as add-on therapy with lactulose for secondary prophylaxis of overt hepatic encephalopathy.^1^

Hepatic encephalopathy, despite initial recovery with treatment results in significant cognitive impairment with manifestations like memory loss, emotional disturbances, behavioral changes, loss of fine motor skills and psychosocial dysfunction.^4^ Quality of life is never the same even after first attack of encephalopathy with poor survival.^3^ Therapeutic intervention with potential to avert first episode, i.e. primary prophylaxis for hepatic encephalopathy can save patient from this disability.

Role of rifaximin for secondary prophylaxis of encephalopathy has extensively been studied in our population with mixed results^7^ and it has shown to be effective even in low dose^8^ but its efficacy for primary prophylaxis of encephalopathy is not much explored. Few studies have shown its benefit but data available is insufficient to recommend its use. Moreover due to high cost and few GI related side effects of rifaximin, efficacy of low dose rifaximin for primary prophylaxis also needs to be determined as compared to full dose. We planned a study to determine efficacy of rifaximin for primary prophylaxis of hepatic encephalopathy and to compare high vs low dose of rifaximin in prevention of hepatic encephalopathy in patients with decompensated liver cirrhosis.

## METHODS

A quasi experimental double blind randomized trial was carried out at Gastroenterology Ward, Medical Unit III, Services Institute of Medical Sciences / Services Hospital, Lahore from August 2017 to August 2018. After approval by Ethical Review Board of SIMS, patients with decompensated liver cirrhosis being admitted in hospital with no overt hepatic encephalopathy at admission or in past according to West-Haven criteria were included.^9^ Decompensated liver cirrhosis was defined as history of or presence of overt clinical evidence of either esophageal varices bleeding, jaundice or ascites in a patient of liver cirrhosis.^10^ Patients under 18 years of age, patients with any type of dementia or manifestations of neuropsychiatric illnesses, those with any bacterial infection at time of admission, patients receiving secondary prophylaxis for spontaneous bacterial peritonitis and those having used rifaximin in previous 6 months were excluded from study.

Sample size was calculated keeping one-sided level of significance of 5%, (α=0.05) statistical power 85% (1-β= 0.085) and on assumption that difference of 10% in outcome of two groups will be acceptable. Calculated sample size was 75 but we increased it to 80 to accommodate possible drop outs in follow up.

Informed consent was obtained from all included patients. Detailed medical history and neurological examination with assessment for West-Haven criteria, was performed on each patient. Laboratory investigations including complete blood count, liver function tests, renal function test, and coagulation profile along with abdominal ultrasound were performed. Patients were randomized before discharge from hospital in 2 groups using online random table generator Stat Trek^®^ by investigator no 2 and 3 (BM, AA). Group-A patients received Tab Rifaximin 220 mg twice a day while Group-B patients were given Tab Rifaximin 550 mg twice a day along with lactulose syrup and other medications as needed for co-morbid issues.

Patients were followed fortnightly in first month and then monthly for 6 months by investigator number one and four (SS, MNA) in outpatient clinic not aware of group identity of patient. On each follow up visit detailed history and examination for overt hepatic encephalopathy (OHE) was carried out. OHE was defined as brain dysfunction caused by liver insufficiency and/or portosystemic shunting and manifests as a wide spectrum of neurological/psychiatric abnormalities ranging from mild clinical alteration to coma and staged as per West Haven Criteria.^11^ Primary end point of study was the development of OHE during follow up time or death of patient.

### Statistical Analysis

Data was analyzed using SPSS® 20.0 (Armonk, NY: IBM corp) Patients were entered as Group-A and B in SPSS not disclosing group identity to statistician. Variable were expressed as mean ± standard deviation (SD) or in percentage where appropriate for normally distributed variables while median and interquartile range (IQR) for variables not normally distributed. Shapiro-wilk test was used for checking whether variables were normally distributed or not.

Unpaired student’s t test was used to compare numerical variables while chi square X^2^ test was used to compare categorical variables. Mann Whitney U test was used for variables not normally distributed. Cox regression analysis was done to determine hazard ratio (HR) and its 95% confidence interval for developing hepatic encephalopathy and mortality. P value of ≤0.05 was considered statistically significant.

## RESULTS

Total of 75 patients were included in final analysis as five patients of Group-A were lost to follow up. Mean age of study patients was 53.8(±10.7) and male to female ratio was 0.97/1 (37/38). All patients had decompensated cirrhosis, 61(81.3%) had history of variceal bleeding while 52 (69.3%) had history of ascites. Ascites was present at inclusion in 48 (64%) patients, mild in 11, moderate in 31 and severe in six patients. Child Pugh Turcotte (CTP) class A disease was present in 12 (16%) patients, class B in 45 (60%) and C in 18 (24%) patients. In final analysis 34 (45.3%) patients of Group-A and 41 (54.7%) patients of Group-B were included. We compared both groups as shown in Table-I. Patients in Group-B had significantly advanced liver disease than Group-A as higher CTP score (p value 0.03) and higher MELD score (p value 0.04) was noted in Group-B.

**Table I:** Comparison of patients in Group-A and B.

Variables	Group-A (n- 34)	Group-B (n- 41)	P value

	Mean(±SD)	Mean(±SD)	
Age (years)	52.7(10.1)	54.8(11.2)	0.38
Platelet (x10^9^/L)	106.6(66.3)	114.5(85.3)	0.65
INR	1.39(0.41)	1.65(0.71)	0.06
Albumin (g/dl)	2.74(0.46)	2.70(0.52)	0.71
Creatinine (mg/dl)	1.3(0.8)	1.5(1.4)	0.48
CTP score	7.76(1.3)	8.63(2.05)	0.03
MELD score	14.3(7.5)	18.1(8.5)	0.04

	*No of patients (n-34)*	*No of patients (n-41)*	

Hematemesis	29	32	0.42
Ascites	22	30	0.42

During 6 month follow up 24 (32%) patients developed hepatic encephalopathy. It was of West Haven stage-I in 2 (2.7%) patients, stage-II in 2 (2.7%), stage-III in 7 (9.3%) and stage-IV in 13 (17.3%) patients. In Group-A 12 (35.2%) patients had encephalopathy during follow up while 12 (29.2%) patients of Group-B developed encephalopathy and difference in two groups was not significant (p value 0.57). We compared patients who developed hepatic encephalopathy and those who did not as shown in Table-II.

**Table II:** Comparison of patients who developed encephalopathy with those who had no encephalopathy in follow up.

Variables	Patients with encephalopathy n- 24	Patients with no encephalopathy n- 51	P value

	Mean± SD	Mean± SD	
Age	56.4(14.3)	52.7(8.4)	0.16
Platelet (x 10^9^/L)	107.1(82.1)	112.7(75)	0.77
INR	1.44(0.4)	1.58(0.67)	0.35
Bilirubin (mg/dl)	4.59(7.1)	1.67(1.64)	0.007
Albumin (g/dl)	2.5(0.4)	2.8(0.51)	0.03
Creatinine (mg/dl)	1.63(1.1)	1.34(1.23)	0.33
CTP score	8.8(1.89)	7.94(1.69)	0.03
MELD score	18.5(9.1)	15.4(7.7)	0.13

During 6 month follow up of study patients, 13 (17.3%) patient died all due to worsening liver disease resulting in liver failure. Death was noted in 6 (17.6%) patients of Group-A and in 7 (17.07%) patients of Group-B. Difference in mortality between two groups was again not significant (p value 0.94). Patients who died of cirrhosis in follow up had higher bilirubin levels (p < 0.00), higher serum creatinine (p 0.05), high CTP score (p 0.04) and higher MELD score (p 0.004).

**Fig.1:**
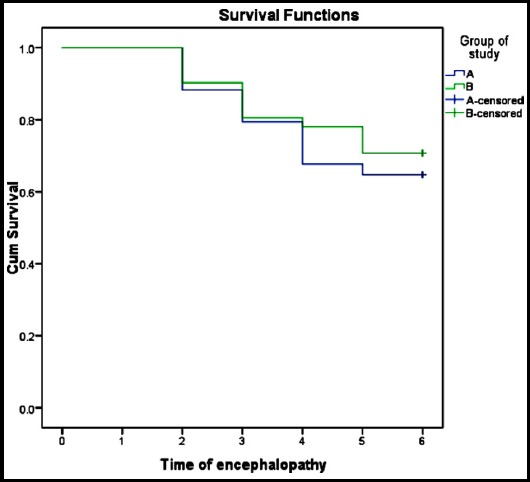
Kaplan Mayer Survival curve comparing Group-A and B.

Hazard ratio for developing encephalopathy was 0.80 (95% CI 0.36-1.78) with two log likelihood of 200.94 suggesting no significant difference in two groups (p value 0.58). Similarly hazard ratio for death was 0.95 (95% CI 0.32-2.85) and 2 log likelihood of 111.12 with p value of 0.94. Kaplan-Mayer Survival curves for both groups are shown in Fig.1.

Side effect profile of rifaximin in study patients revealed bloating and abdominal pain in 13 (17.3%) patients, nausea/vomiting in 7 (9.3%) patients and headache in 9 (12%) patients. No difference in side effects was seen among low dose vs high dose rifaximin.

## DISCUSSION

Liver cirrhosis, as long as it is compensated is associated with good quality of life and good prognosis. Once decompensated, its progress to stage of liver transplantation or death is accelerated.^12^ Liver decompensation not only results in poor quality of life but is also responsible for recurrent hospital admissions, employment disability, impaired fitness for driving and unbearable economic burden for both family and health system. In a study by Bajaj JS et al, once decompensated, three months re-admission in hospital was 53% and leading cause for this was hepatic encephalopathy.^13^ One episode of OHE is associated with 40% risk of recurrence in next six months.^14^ It was thought that no residual neurocognitive impairment is left after recovery from an episode of OHE. Recent studies have found persistent and cumulative deficits in working memory and learning abilities of patient even after one episode of OHE.^15^ Poor prognosis and psychosocial effects of hepatic encephalopathy calls for efforts to prevent this complication.

Patients with cirrhosis has up to 25% chances of developing OHE in 5 years but once liver is decompensated then overall median survival is two years and risk of OHE varies between 20-40% in a year depending on presence of risk factors like ascites or variceal bleeding.^14^ Effective primary prophylaxis should bring down risk of OHE in decompensated liver disease. In our study with rifaxamin use primary OHE developed in 32% patients and mortality was 17.2% in six month follow up. This efficacy is inferior as compared to lactulose which in a landmark study by Sharma P et al reduced chances of first episode of OHE to 11% in 1 year as compared to 28% in control group.^16^ Relatively high rate of OHE in study patient even with rifaximin and lactulose is probably due to advanced stage disease of study population as 31 (41.3%) of our patients had MELD score of 18 or above.

Sidhu SS et al evaluated effect of rifaximin in patients with chronic hepatic encephalopathy with no episode of OHE and concluded that it results in improvement of cognitive functions in 75% of patients as compared to only 20% improvement in those not receiving rifaximin.^17^

Higuera-de-la-Tijera F et al compared lactulose, rifaximin and L-ornithine L-aspartate for primary prophylaxis of OHE in patients with variceal bleeding. OHE was seen in 54.5% patients of placebo group, 27.3% in lactulose group, 22.7% in L-Ornithine L-aspartate Group-And 23.8% in rifaximin group with OR of 0.3, 0.2 and 0.3 respectively.^18^ They concluded rifaximin and other anti-ammonium medications to be effective in primary prophylaxis of OHE in variceal bleeding. In a systematic review with meta-analysis regarding effects of rifaximin in hepatic encephalopathy, Kimer N et al concluded that rifaximin is effective in prevention of OHE with RR 1.36; (95% CI 1.06-1.65)^19^ Despite these few promising studies, leading hepatology societies still don’t recommend rifaximin for primary prophylaxis of OHE due to weak quality of evidence.^10^ Performance of rifaximin in our study for primary prophylaxis of OHE was also suboptimal.

No difference in efficacy was seen in low dose Rifaximin group vs. high dose in prevention of OHE. Similarly side effects of drug in study patients were minor, mostly GI related which can easily be managed. Muller KD et al evaluated efficacy of rifaximin 100mg/day use for more than two years and concluded that it is very effective in prophylaxis of OHE and is safe with no major side effect for long term use.^20^ Due to lesser cost of low dose rifaximin, it may be preferred for use in primary prophylaxis of OHE.

As 32% patients developed OHE with rifaximin in our study, which is not optimum when compared to lactulose alone which is already recommended for primary prophylaxis of OHE, routine use of rifaximin as primary prophylactic drug still can’t be recommended. More and more data regarding efficacy of rifaximin and search for newer drugs for averting this catastrophic complication of liver cirrhosis is needed in this era of direct acting antiviral drugs where patients of decompensated cirrhosis, after viral clearance with antiviral therapy, are expected to live longer without liver transplantation.

## CONCLUSION

Rifaximin use for primary prophylaxis of overt hepatic encephalopathy is not effective in patients with decompensated cirrhosis.

### Authors’ Contributions

**SS:** Conceived, designed, data collection, statistical analysis and manuscript writing

**BM, AA:** Ethical Approval, data collection and manuscript review

**MAN:** Manuscript review and final approval.
